# Neuroprotective Effects of a Smoothened Receptor Agonist against Early Brain Injury after Experimental Subarachnoid Hemorrhage in Rats

**DOI:** 10.3389/fncel.2016.00306

**Published:** 2017-01-18

**Authors:** Quan Hu, Tong Li, Lingxiao Wang, Yunkai Xie, Song Liu, Xuemei Bai, Tiantian Zhang, Shishi Bo, Danqing Xin, Hao Xue, Gang Li, Zhen Wang

**Affiliations:** ^1^Department of Neurosurgery, Qilu Hospital of Shandong University and Brain Science Research Institute, Shandong UniversityJinan, China; ^2^Department of Physiology, Shandong University School of MedicineJinan, China; ^3^Department of Neurosurgery, Taian Central HospitalTaian, China

**Keywords:** purmorphamine, Shh signaling, subarachnoid hemorrhage, early brain injury, oxidative stress

## Abstract

The sonic hedgehog (Shh) signaling pathway plays a fundamental role in the central nervous system (CNS) development, but its effects on neural cell survival and brain repair after subarachnoid hemorrhage (SAH) has not been well-investigated. The present study was undertaken to evaluate the influence of an agonist of the Shh co-receptor Smoothened (Smo), purmorphamine (PUR), on early brain injury (EBI) as well as the underlying mechanisms after SAH. PUR was administered via an intraperitoneal injection with a dose of 0.5, 1, and 5 mg/kg at 2, 6, 24, and 46 h after SAH in rat model. The results showed that PUR treatment significantly ameliorated brain edema, improved neurobehavioral function, and attenuated neuronal cell death in the prefrontal cortex (PFC), associated with a decrease in Bax/Bcl-2 ratio and suppression of caspase-3 activation at 48 h after SAH. PUR also promoted phospho-ERK levels. Additionally, PUR treatment markedly decreased MDA concentration accompanied with the elevation in the expression of nuclear factor erythroid 2-related factor 2 and heme oxygenase-1 in PFC. Notably, PUR treatment significantly reversed the changes of Shh pathway mediators containing Patched, Gli1, and Shh by SAH insult, and the neuroprotection of PUR on SAH was blocked by Smo antagonist cyclopamine. These results indicated that PUR exerts neuroprotection against SAH-evoked injury in rats, mediated in part by anti-apoptotic and anti-oxidant mechanism, up-regulating phospho-ERK levels, mediating Shh signaling molecules in the PFC.

## Introduction

Increasing body of clinical and experimental data has demonstrated that early brain injury (EBI) largely contributes to unfavorable outcomes after subarachnoid hemorrhage (SAH; Bederson et al., 2009). Many studies have shown that elevation of intracranial pressure, oxidative stress, cerebral perfusion disruption, blood-brain barrier (BBB) damage, cerebral edema, and cell apoptosis contributed to the development of EBI, however, the exact mechanism of EBI remains elusive (Sehba et al., 2012).

The sonic hedgehog (Shh) signaling pathway plays a fundamental role in the central nervous system (CNS) development. Shh is a morphogenic protein that binds to its receptor, Patched (Ptch), on the surface of target cells, and cause Ptch activation. Ptch activation releases Smoothened (Smo) to initiate downstream signaling that controls the transcription factor Gli-1. Then Gli-1 translocates to the nucleus and thereby regulates the expression of a series of target genes that control cell growth, survival, and differentiation (Chari and McDonnell, 2007). Recent studies showed the beneficial role of Shh signaling in various models in the process of neurodegenerative diseases and brain injury, including acute brain injury (Amankulor et al., 2009), Parkinson's disease (Tsuboi and Shults, 2002), stroke (Huang et al., 2013; Chechneva et al., 2014), multiple sclerosis and demyelination (Franco et al., 2008), spinal cord injury (Bambakidis et al., 2010), HIV-associated neurological disorders (Singh et al., 2016). Moreover, using direct Shh protein injection, such as administration of recombinant Shh protein or intravenous hedgehog agonist, Ag11.1, was reported to improve spinal cord injury in adult rats (Bambakidis et al., 2003, 2010). The administration of Shh agonist, SAG, can alleviate HIV-associated neuropathology (Singh et al., 2016). Purmorphamine (PUR) is a purine-derivative small molecule agonist of SMO receptors (Sinha and Chen, 2006). In 2002, Schultz et al. were the first to demonstrate that PUR could induce osteoblast differentiation of multipotent mesenchymal progenitor cells (Wu et al., 2002). In addition, PUR increases resistance of hippocampal neurons against oxidative challenge and reduces neuronal death (Peterson and Turnbull, 2012). The protective role of PUR was reported to promote BBB formation and act as an endogenous anti-inflammatory system (Alvarez et al., 2011). However, the neuroprotective effects of PUR on EBI have not been investigated. In the present study, we aimed to investigate the role of PUR in EBI post-SAH insult and the underlying mechanism by employing an experimental SAH rat model.

## Materials and methods

### Establishing SAH model

All the procedures were proved by the Ethic Committee of Medical Department, Shandong University and Qilu Hospital which conformed to the International Guiding Principles for Animal Research, as stipulated by the World Health Organization (Howard-Jones, 1985).

Adult male Wistar rats weighting 280–350 g were obtained from Laboratory Animal Center, Shandong University and then were housed under temperature- and light-controlled laboratory conditions with free access to food and water for 7 days. Experimental animals were subjected to SAH by double blood injection method according to previous study (Li et al., 2016). Firstly, deep anesthesia was induced under 3.5% isoflurane and maintained the anesthesia state at a concentration of 2.5%. Then 200 μl autologous blood was withdrawn from the femoral artery and given into the cisterna magna at a speed of 50 μl per min.

### Drug treatment and groups design

Total 162 rats were used in the study. All the rats were randomly assigned to six groups: sham+vehicle (saline) group, the SAH+vehicle (saline) group, the SAH+PUR1 (0.5 mg/kg) group, the SAH+PUR2 (1 mg/kg) group, the SAH+PUR3 (5 mg/kg) group, and the SAH+PUR (1 mg/kg)+cyclopamine (Cyc, 1 mg/kg). Drug or saline was intraperitoneally given at 2, 6, 24, and 46 h post-SAH. At 48 h after SAH, the animals were sacrificed for tissue analysis.

### Neurological assessment

Neurological examinations of scoring system (Table 1) were blindly performed at 48 h after SAH according to previous research (Cui et al., 2015), monitoring three aspects: appetite, activity, and neurological deficits. Neurological deficits of the experimental animals were scored as follows: (i) “no neurologic deficit” scored as 0; (ii) “potential or minimum neurologic deficit” scored as 1; (iii) “mild neurologic deficits” scored as 2 or 3; (iv) “severe neurologic deficit” scored as 4–6.

**Table 1:** **Behavior and activity scores**.

**Category**	**Behavior**	**Score**
Appetite	Finished meal	0
	Left meal unfinished	1
	Scarcely ate	2
Activity	Walk and reach at least three corners of the cage	0
	Walk with some stimulations	1
	Almost always lying down	2
Deficits	No deficits	0
	Unstable walk	1
	Impossible to walk	2

### Brain water content

Brain edema was evaluated using the wet/dry method which was calculated as [(wet weight − dry weight)/wet weight] × 100%. Brain samples were rapidly removed and weighed immediately as wet weight at 48 h after SAH. They were subsequently dried for 48 h at 100°C and weighed as the dry weight.

### Morphological analysis

The brains were removed quickly on ice and fixed in formalin. After fixation and dehydration in gradient ethanol, the tissues were embedded in paraffin and sliced into 4 μm thickness sections using a section cutter (Leica, Germany). Three sections/rat were stained with hematoxylin and eosin (H&E). Morphological observation of prefrontal cortex (PFC, the cerebral cortex which covers the front part of the frontal lobe) was obtained from three sections per rat by a light microscope (Olympus Corporation, Japan). Each group provided four rats for HE staining.

Injured neurons were identified by their acidophilic (eosinophilic) cytoplasm and pyknotic nuclei with HE staining (Medel-Matus et al., 2014). Three microscope areas (40×) in PFC were obtained and counted damaged cells by a blind manner. Average of the number of injured cells was obtained from three sections per rat. For each selected field, only cells with their nuclei present in the focal plane were counted.

Other sections were used to demonstrate the apoptotic cells in PFC by terminal deoxynucleotidyl transferase dUTP nick end labeling (TUNEL) which was operated according to the manufacturer's protocol (Dead End Flurometric Kit, Promega, WI, USA). Three rats for each group were prepared to paraffin sections for the staining. The slides were counter-stained with 4′,6-diamidino-2-phenylindole (DAPI). Three microscope areas (20×) of TUNEL-positive cells in PFC were observed and imaged by a researcher who was blind to the condition. TUNEL/DAPI positive cells were counted as the mean of the numbers from the six pictures/rat.

### Immunofluorescence staining of cleaved caspase-3

Procedures on immunofluorescence staining to detect caspase-3 activation were as follows: sections were dewaxed with a standard procedure and washed with PBS. Then the sections were incubated at 4°C for 12 h overnight with the primary antibody (cleaved caspase-3, 1:100, Cell Signaling Tech. MA, USA; andNeuN, 1:100, Abcam, Cambridge, MA, USA), followed by the appropriate fluorescent-conjugated secondary antibody (1:500, Sigma-Aldrich). Three microscope fields (20×) of active caspase-3/NeuN double positive cells in PFC were obtained using a Nikon TE2000U microscope by a blind manner. Active caspase-3/NeuN double positive cells were recorded as the mean of the numbers from six pictures/rat. Each group provided four rats for the staining.

### Malondialdehyde (MDA) content

The tissues from PFC (six mice for each groups) were homogenized with tissue protein extraction kits (Pierce Biotechnology, Inc., IL, Rockford, USA) including protease inhibitors and then centrifuged at 12,000 g for 10 min. The supernatant was prepared for subsequent analyses of MDA content, which was assessed with a multiwell spectrophotometer (Bio-Rad, USA). Measurement of MDA content mainly depends on the production of lipid peroxidation and was detected using an assay kit (Jiancheng Inc., Nanjing, China) by thiobarbituric acid analysis (Draper and Hadley, 1990). The evaluations were determined by standard protocols and normalized to the protein content.

### Reverse transcription-polymerase chain reaction (RT-PCR)

Total RNA was extracted from the PFC tissue through the Trizol reagent (Gibco, Invitrogen) method. The concentration of total RNA was then detected by a spectrophotometer (Bio-Rad. Labs) at 260 nm. Total 2 μg of RNA were reversely transcribed into cDNA using a RT-PCR kit (Fermentas, Vilnius, Lithuania) and then was amplified by PCR with specific primers (Table 2). The products were separated on a 1.2% agarose/TAE gel and stained with ethidium bromide to visualize. β-actin was recorded as the internal control. Data on the intensity of bands was collected and analyzed by Image-Pro Plus 6.0 software.

**Table 2:** **PCR primers used in this study**.

**Gene**	**Forward (5′ →3′)**	**Reverse (5′ →3′)**
Bax	GGT TGC CCT CTT CTA CTT TGC	TCT TCC AGA TGG TGA GCG AG
Bcl-2	GGA TGA CTT CTC TCG TCG CTA C	TGA CAT CTC CCT GTT GAC GCT
Shh	AAG CTG GTG AAG GAC TTA CG	AAA GAG CGC GCT TGG C
Gli-1	TAG ATG AAG CTC AAG GGC TG	TAG CCA TTTA GGA GAC GTG G
Ptch	TGG CCT CGG CTG GTA AC	ATA TGA GGA GAC CCA CAA CC
β-actin	CTA TTG GCA ACG AGC GGT TCC	CAG CAC TGT GTT GGC ATA GAG G

### Western blot analysis

Protein concentration in the PFC was determined as reported previously. Equal quantities of protein were loaded onto a 10–15% gradient polyacrylamide gel, electrophoretically transferred to a polyvinylidene difluoride membrane and probed with the following primary antibodies: Bcl-2 antibody (1:1000, Santa Cruz Biotechnology, CA, USA), Bax antibody (1:1000, Santa Cruz Biotechnology), Shh (1:1000, Proteintech.), Gli-1 (1:1000, Santa Cruz Biotechnology), Ptch (1:1000, Abcam, Cambridge, MA, USA), caspase-3 (1:500, Cell Signaling Tech. MA, USA), cleaved caspase-3 (1:1000, Cell Signaling), Phospho-extracellular signal-regulated kinase (ERK)1/2 (1:2000, Cell Signaling), ERK1/2 (1:2000; Cell Signaling), Nrf2 (1:1000, Proteintech.), HO-1 (1:2000, Proteintech.). β-actin (1:2000; Sigma-Aldrich) was used as an internal loading control.

### Statistical analysis

Values were expressed as the mean ± SD. Significant difference was performed with a one-way ANOVA using the *post-hoc* Tukey-test for multiple comparisons of means. *p* < 0.05 was considered to be a significant difference.

## Results

### Influence of PUR on mortality, neurological deficits, and edema after SAH

**Figure 1:**
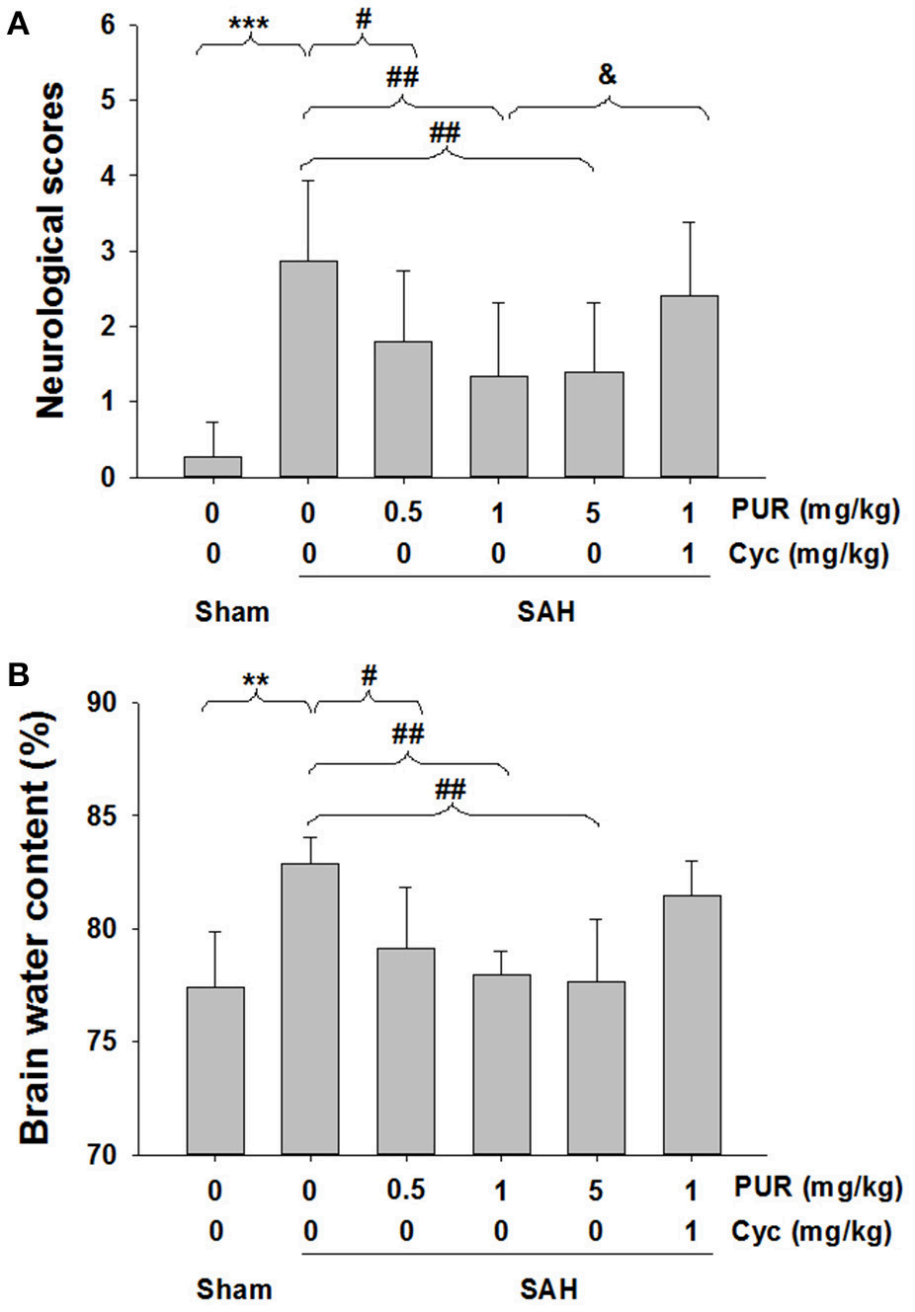
**PUR attenuated SAH-induced neurological deficits and brain edema. (A)** Neurological scores were recorded at 48 h after SAH, *n* = 15. **(B)** Brain water content of cerebral cortex was measured at 48 h after SAH, *n* = 6. Values represent the mean ± SD. ^**^p < 0.01, ^***^p < 0.001 SAH vs. Sham, ^#^*p* < 0.05, ^##^*p* < 0.01 SAH+PUR vs. SAH, ^&^*p* < 0.05 SAH+PUR+Cyc vs. SAH+PUR.

Total 162 rats were used for surgeries. And 18 rats died within 1 h after surgery (18/162 operated animals) during which time the animals had not received either the drug or the vehicle yet. Within 48 h after surgeries, the mortality in each group was: sham 0% (0 of 20), SAH+vehicle 17.86% (5 of 28), SAH+PUR1 12.5% (3 of 24), SAH+PUR2 8.33% (2 of 24), SAH+PUR3 4.16% (1 of 24), SAH+PUR3+Cyc 16.67% (4 of 24). A Pearson chi-squared analysis revealed that treatment with PUR or Cyc has no significantly different in mortality as compared to vehicle treatment in SAH rats (*p* > 0.05) (Table 3). As shown in Figure 1A, the neurological deficits in SAH groups increased significantly compared to the sham group (*p* < 0.001). However, PUR treatment at 0.5, 1 and 5 mg/kg reduced neurological deficits compared with those in the SAH+vehicle group after 48 h SAH (*p* < 0.05, *p* < 0.01, *p* < 0.01, respectively). Co-treatment with Cyc, a SMO antagonist, suppressed this effect compared to SAH+PUR.

**Table 3:** **Demographic information of SAH rats in different treatment (total sample size: ***n*** = 120)**.

	**Group**				
**Characteristics**	**SAH+Vehicle**	**SAH+PUR**	**SAH+PUR+Cyc**	**Total number**	***p-*****Values**
Dead number	5	6	4	15	<0.05^#^
Survival number	23	72	20	115	
Total	28	78	24	120	

# *Pearson Chi-Squared test*.

As shown in Figure 1B, the brain water content was markedly elevated (*p* < 0.01) in SAH groups in comparison with the sham group, while after PUR administration at 0.5, 1, and 5 mg/kg, the brain edema was dramatically attenuated (*p* < 0.05, *p* < 0.01, *p* < 0.01, respectively).

### PUR alleviates SAH-induced brain injury

**Figure 2:**
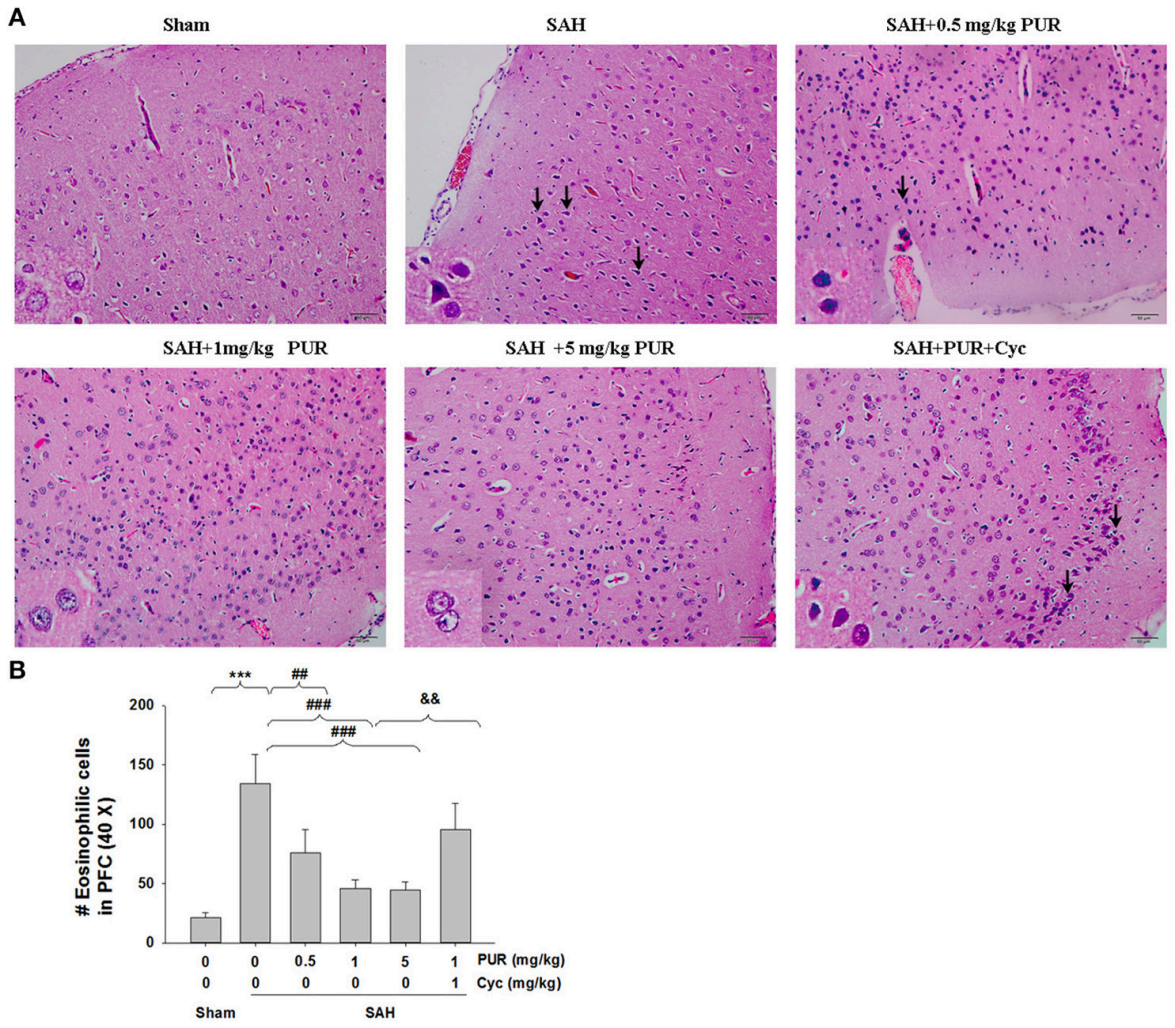
**PUR ameliorated SAH-induced brain injury. (A)** HE staining of the brain tissues was taken at 48 h after SAH. Pathological changes represent focal edema and neuronal cell death (marked by black arrow) in the prefrontal cortex (PFC). The high magnification shows representative normal or damaged cells, *n* = 4. Scale bar = 50 μm. **(B)** Bar graphs showing quantification of eosinophilic cells with pyknotic nuclei detected in the PFC, *n* = 4. Values represent the mean ± SD. ^***^p < 0.001 SAH vs. Sham, ^##^*p* < 0.01, ^###^*p* < 0.001 SAH+PUR vs. SAH, ^&&^*p* < 0.01 SAH+PUR+Cyc vs. SAH+PUR.

As shown in Figure 2, HE staining showed that the PFC in the sham group has clear structural layers and neurons presented clear borderline. While in SAH group, cells were arranged sparsely, and the cell outline was fuzzy. Moreover, neurons were shrunken, and obvious edema was found in the PFC which was pale, in SAH group. Treatment with PUR in SAH reduced brain edema and this morphological damage (Figure 2).

**Figure 3:**
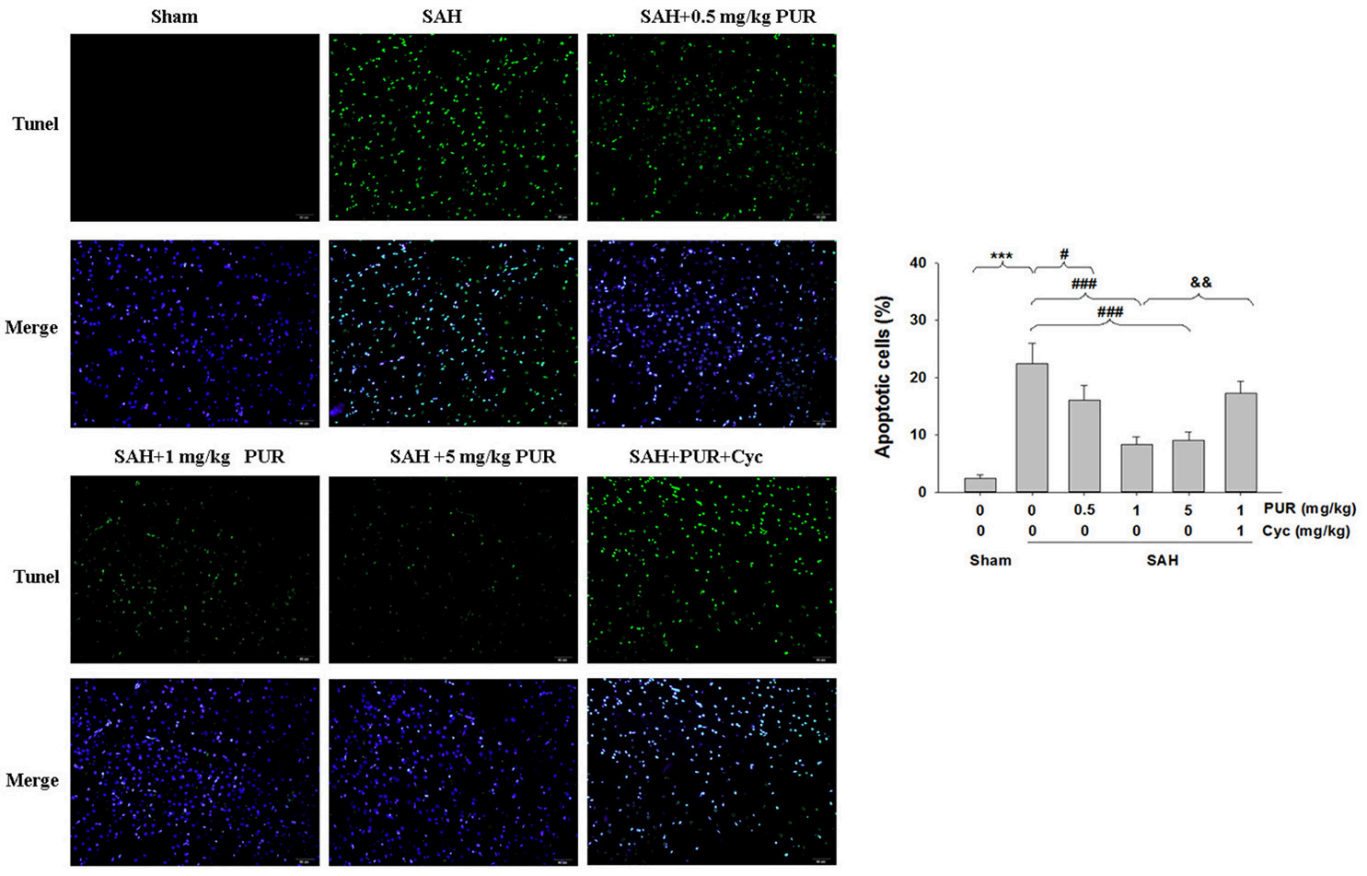
**PUR attenuates SAH-induced apoptosis**. The detection of TUNEL-positive cells in PFC was taken at 48 h after SAH. Scale bar = 50 μm. Bar graphs showing quantification of TUNEL-positive cells, *n* = 3. Values represent the mean ± SD. ^***^p < 0.001 SAH vs. Sham, ^#^*p* < 0.05, ^###^*p* < 0.001 SAH+PUR vs. SAH, ^&&^*p* < 0.01 SAH+PUR+Cyc vs. SAH+PUR.

The TUNEL staining showed that apoptotic cells were rare in the PFC in the sham group, while that TUNEL-positive cells in PFC were significantly increased at 48 h after SAH insult (*p* < 0.001). Administration of PUR at 0.5, 1, and 5 mg/kg significantly decreased the TUNEL-positive neurons (*p* < 0.05, *p* < 0.001, *p* < 0.001, respectively, Figure 3) in comparison with the SAH+vehicle group. Moreover, Co-treatment with Cyc increased TUNEL-positive cells compared to SAH+PUR group.

### The effect of PUR on caspase-3 activation after SAH

**Figure 4:**
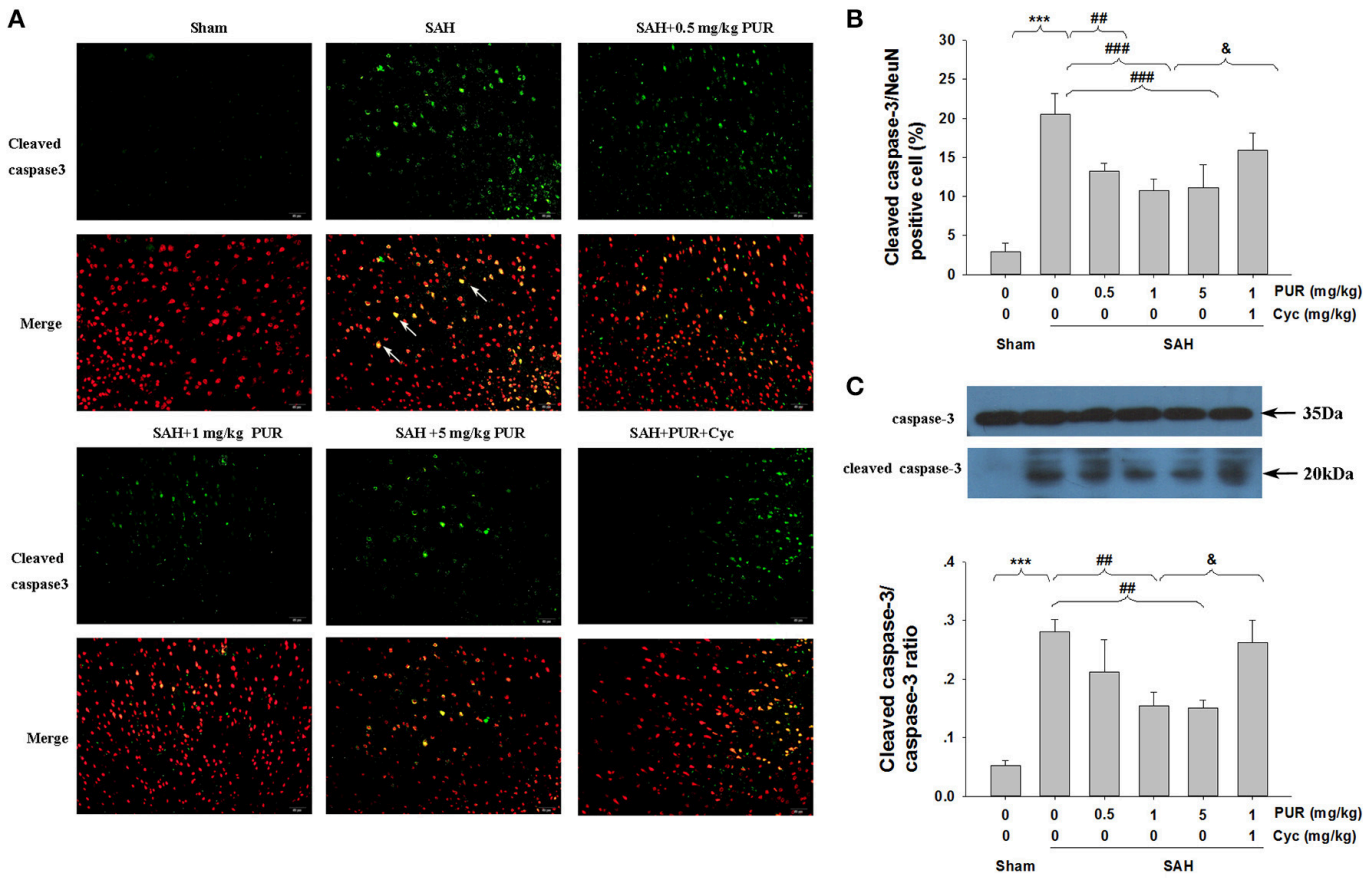
**The effect of PUR on caspase-3 activation in SAH. (A)** Immunofluorescene study showed double stating of active caspase-3 (green) and NeuN (red) of PFC at 48 h after SAH. Scale bar = 50 μm. **(B)** Bar graphs showing quantification of active caspase-3/NeuN-positive cells, *n* = 4. **(C)** The expression of cleaved caspase-3 was assessed using western blot analysis. Bar graphs showing quantification of the protein levels of cleaved caspase-3 and caspase-3 were determined by Image-Pro Plus 6.0. Results were expressed as cleaved caspase-3/caspase-3 ratio, *n* = 3. Values represent the mean ± SD. ^***^p < 0.001 SAH vs. Sham, ^##^*p* < 0.01, ^###^*p* < 0.001 SAH+L-Cys vs. SAH, ^&^*p* < 0.05 SAH+PUR+Cyc vs. SAH+PUR.

NeuN is a neuronal-specific nuclear protein, which is expressed in most neurons in the brain. To investigate the potential protective mechanism of PUR, we performed active caspase-3/NeuN staining after SAH. Figure 4 showed that numerous active caspase-3/NeuN double positive-stained neurons were observed in the PFC of SAH group, when compared with the sham rats (*p* < 0.001). However, intervention with PUR at 0.5, 1, and 5 mg/kg significantly reduced the active caspase-3/NeuN double positive-stained neurons in SAH group (*p* < 0.05, *p* < 0.05, *p* < 0.05, respectively, Figures 4A,B), compared with the vehicle-treated SAH group.

In the SAH group, the level of cleaved caspase-3 expression in the PFC were significantly increased (*p* < 0.001, Figure 4C) when compared with the sham rats by Western blot analysis. Co-treatment with PUR at 1 and 5 mg/kg reduced (*p* < 0.01, *p* < 0.01, respectively) the level of cleaved caspase-3 expression (Figure 4C), as compared with the SAH groups. And co-treatment with Cyc reversed this effect compared to SAH+PUR.

### Influence of PUR on SAH-induced changes of Bcl-2 and Bax

**Figure 5:**
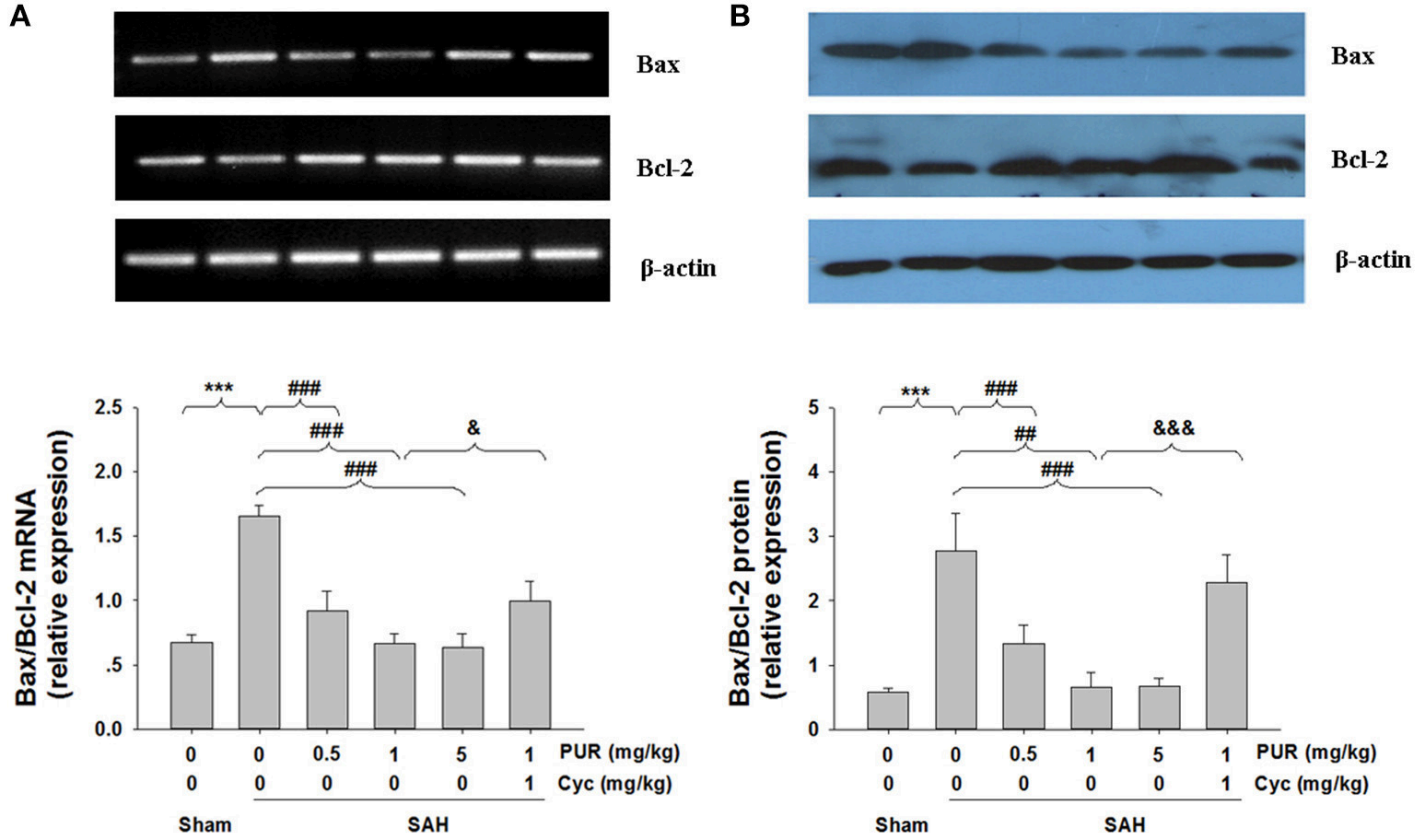
**Effects of treatment with PUR on Bax, Bcl-2 in mRNA and protein levels. (A)** The relative expression levels of Bax and Bcl-2 mRNA in the PFC were analyzed by RT-PCR. The densities of the protein bands were analyzed and normalized to β-actin, *n* = 3. **(B)** Representative western blots showing levels of Bax and Bcl-2 in the PFC, Bar graphs showing quantification of the protein levels of Bax and Bcl-2, *n* = 3. Both the determination of the two signs in mRNA and protein levels were obtained from three separate experiments. Values represent the mean ± SD. ^***^p < 0.001 SAH vs. Sham, ^##^*p* < 0.01, ^###^*p* < 0.001 SAH+PUR vs. SAH, ^&^*p* < 0.05, ^&&&^*p* < 0.001 SAH+PUR+Cyc vs. SAH+PUR.

To further confirm the anti-neuronal apoptotic effects of PUR against SAH insult, the expressions of Bcl-2 and Bax were examined by Western blotting and RT-PCR. As shown in Figure 5, the expression of Bcl-2 was significantly down-regulated, while the expression of Bax was up-regulated after SAH insult. The ratio of Bax/Bcl-2 ratio expression was dramatically increased both at mRNA and protein levels at 48 h after SAH insult. However, PUR treatment almost completely reversed the decreasing of Bcl-2 and Bax expression, as well as the altered ratio of Bax/Bcl-2. Co-treatment with Cyc suppressed this effect compared to SAH+PUR groups.

### PUR stimulates ERK1/2 hosphoryation after SAH

**Figure 6:**
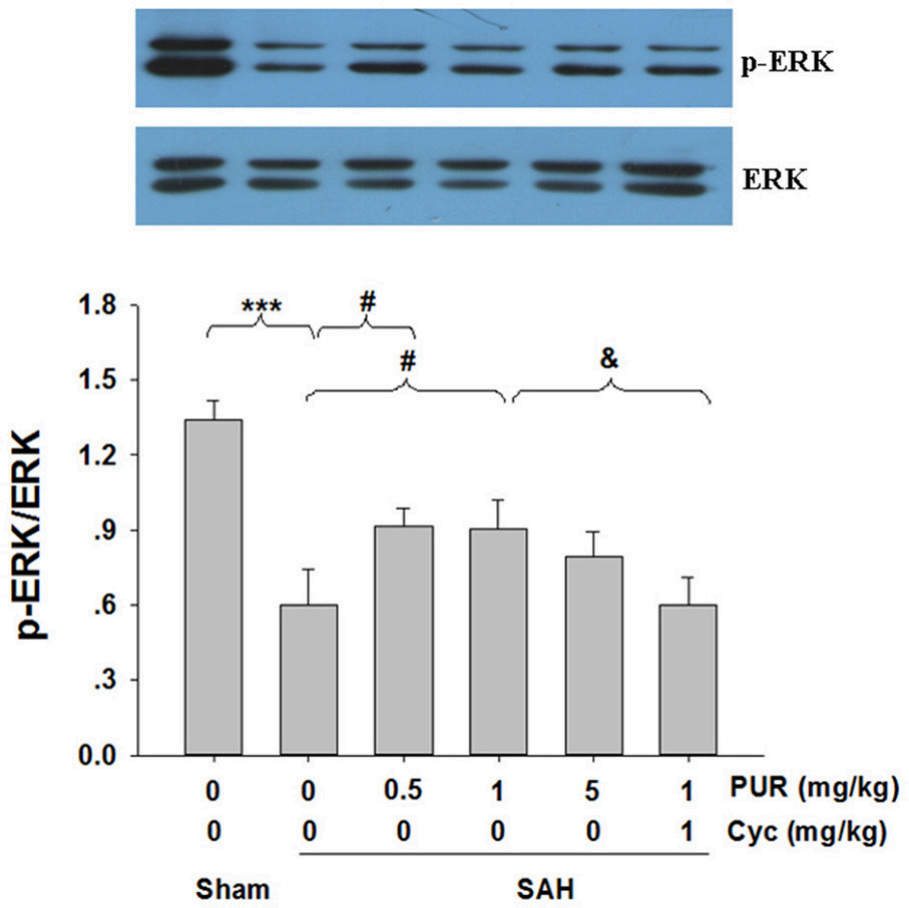
**The effect of PUR on the expression of ERK activation**. At 48 h after SAH, entire PFC extracts were subjected to western blot analysis using an antibody against p-ERK and ERK. Bar graphs showing quantification of expression levels of p-ERK/ERK were determined by the Image-Pro Plus 6.0, *n* = 3. Values represent the mean ± SD. ^***^p < 0.001 SAH vs. Sham, ^#^*p* < 0.05 SAH+PUR vs. SAH, ^&^*p* < 0.05 SAH+PUR+Cyc vs. SAH+PUR.

The roles of ERK1/2 pathways upon the neuroprotective effects of PUR were assessed using Western blot analysis. As shown in Figure 6, SAH insult significantly decreased (*p* < 0.001) the level of phosphorylation of ERK1/2. The intervention with PUR (0.5 and 1 mg/kg) dramatically increased (*p* < 0.05, *p* < 0.05, respectively) the level of phosphorylation of ERK1/2 in the SAH group. And Co-treatment with Cyc inhibited the effect of PUR on ERK1/2 activation.

### Effects of the PUR on MDA levels and Nrf2/ HO-1 expression after SAH

**Figure 7:**
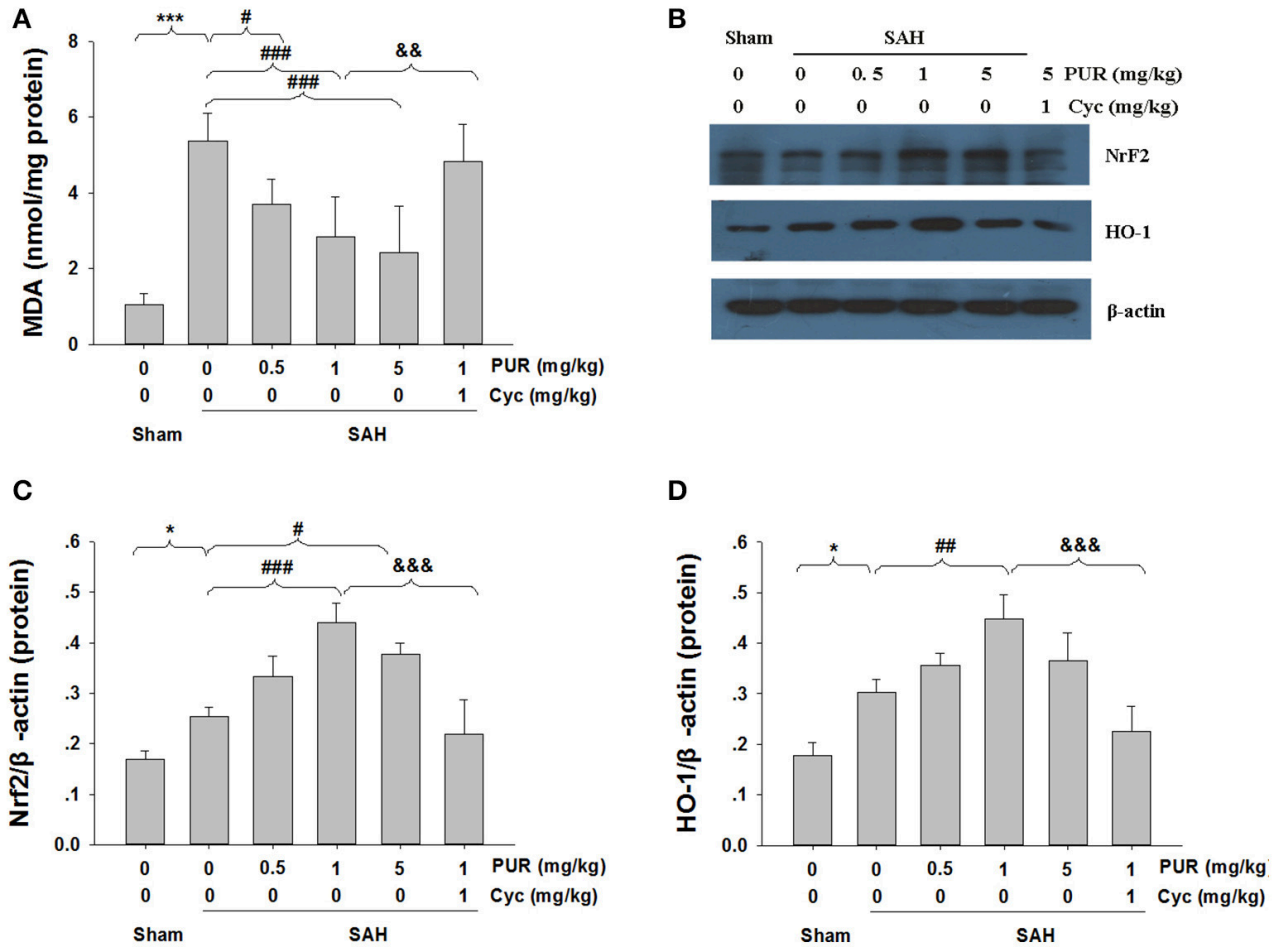
**Effects of the PUR on MDA levels and Nrf2 and HO-1 Expression after SAH. (A)** At 48 h after SAH, entire PFC extracts were subjected to MDA analysis using a commercial kit, *n* = 6. Values represent the mean ± SD. **(B)** At 48 h after SAH, entire PFC extracts were subjected to western blot analysis using an antibody against Nrf2 and HO-1. **(C,D)** Bar graphs showing quantification of levels of Nrf2 and HO-1 were determined by Image-Pro Plus 6.0, *n* = 3. ^*^p < 0.05, ^***^p < 0.001 SAH vs. Sham, ^#^*p* < 0.05, ^##^*p* < 0.01, ^###^*p* < 0.001 SAH+L-Cys vs. SAH, ^&&^*p* < 0.05, ^&&&^*p* < 0.05 SAH+PUR+Cyc vs. SAH+PUR.

MDA can result in tissues and cells damage, thus the content of MDA reflects the level of ROS (Wu et al., 2015). As shown in Figure 7A, the MDA levels in SAH group were significantly increased (*p* < 0.05) as compared to the sham rats. The intervention with PUR at 0.5, 1, and 5 mg/kg significantly decreased (*p* < 0.05, *p* < 0.001, *p* < 0.001, respectively) the level of MDA in the SAH group

As is shown in Figure 7B, Western blot analysis showed that the expressions of Nrf2 and HO-1 were increased in the SAH group, as opposed to the sham group. Moreover, PUR administration further increased the levels of Nrf2 and HO-1 in the SAH group. Co-treatment with Cyc increased MDA levels and down-regulated the expressions of Nrf2 and HO-1 compared to SAH+PUR group.

### Effects of the PUR on Shh signaling

**Figure 8:**
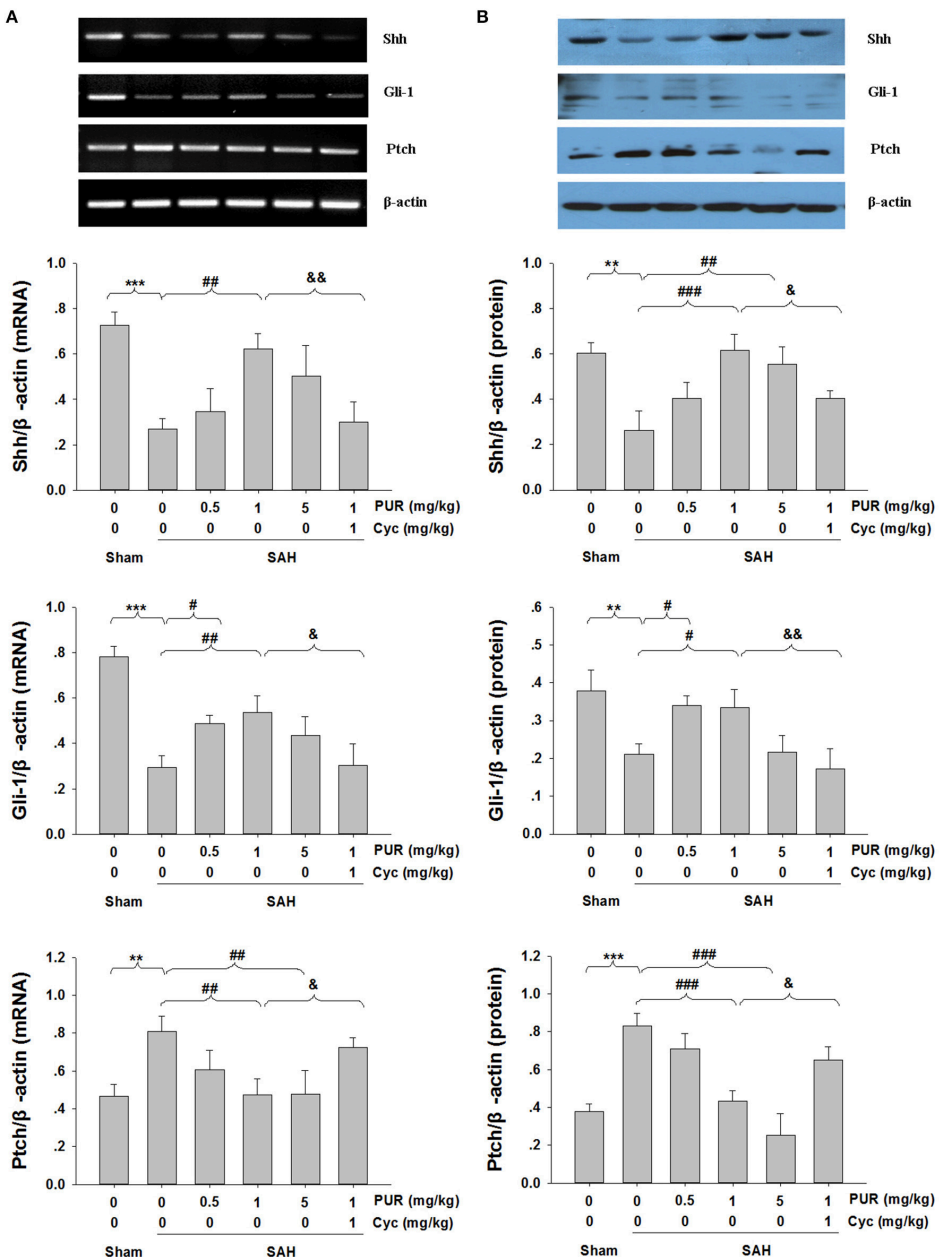
**Effects of PUR on Shh pathway**. The quantification of Shh, Gli-1, and Ptch was measured by RT-PCR **(A)** and western blot **(B)** 48 h post-SAH. Each value was normalized to β-actin. Bar graphs showing quantification of mRNA and protein levels of Shh, Gli-1, and Ptch were determined by Image-Pro Plus 6.0, *n* = 3. Values represent the mean ± SD. ^**^p < 0.01, ^***^p < 0.001 SAH vs. Sham, ^#^*p* < 0.05, ^##^*p* < 0.05, ^###^*p* < 0.001 SAH+PUR vs. SAH, ^&^*p* < 0.05, ^&&^*p* < 0.01 SAH+PUR+Cyc vs. SAH+PUR.

We next evaluated the involvement of Shh signaling molecules in the anti-apoptotic activity of PUR by examining their expression in the PFC at 48 h after treatment. As shown in Figure 8, the mRNA and protein levels of Shh and Gli-1 were markedly decreased after SAH injury, while PUR administration significantly increased the levels of Shh and Gli-1 in the SAH group. In addition, PUR administration reversed the SAH-induced Ptch expression. And Co-treatment with Cyc suppressed this effect compared to SAH+PUR.

## Discussion

Accumulating evidence has shown that EBI largely contributes to poor outcomes of the patients surviving SAH (Bederson et al., 2009), thus effective treatment of EBI may improve the clinical prognosis of patients surviving SAH. In the present study, PUR treatment attenuated EBI in rats following SAH, for example, inhibiting cell death and attenuating brain edema, as well as improving neurological deficits and mortality. Moreover, PUR decreased MDA levels and up-regulated NrF2 and HO-1 levels in PFC post-SAH, associated with affecting the expression of Shh pathway protein. The effects initiated by PUR were significantly abrogated by Smo antagonist administration.

Many studies suggested that Shh pathway played an important role in the development of neurodegenerative diseases and brain injury (Dai et al., 2011; Huang et al., 2013; Chechneva et al., 2014; Chen et al., 2015). Recently, PUR, as an agonist of potential oncogenic Shh signaling, exhibits anti-apoptotic effect, and promotes tissue regeneration after ischemic stroke (Chechneva et al., 2014). In the present study, PUR significantly attenuated brain edema, improved neurobehavioral function, and ameliorated neuronal cell death in the PFC, suggesting PUR plays protective effects in SAH development. With regard to neuroprotection of PUR, there exists only limited information on this topic which consists of data demonstrating that PUR has anti-apoptotic, anti-inflammatory, and pro-angiogenic effects in the ischemic cortex after ischemic insult (Chechneva et al., 2014; Chechneva and Deng, 2015). Apoptosis is considered to be one of the most crucial factors that can exacerbate EBI after SAH. Moreover, damaged neurons following SAH contribute to delayed neurological outcomes (Sabri et al., 2008). ERK pathway controls various cell responses, such as proliferation, differentiation, motility, survival, and metabolism (White et al., 2000; Rauch et al., 2011). ERK phosphorylation can protect cells from apoptosis via transcriptional regulation of anti-apoptotic protein, such as the Bcl-2 and Mcl-1. ERK phosphorylation also results in the phosphorylation of the pro-apoptotic Bim protein (McCubrey et al., 2007), and then prevents Bax activation. The previous studies reported that the phosphorylation of ERK was decreased in brain after SAH insult and increase of its expression was associated with ameliorating neuronal cell death (Lin et al., 2009; Li et al., 2016). In agreement with these studies, we found that SAH insult decreased p-ERK level, while treatment with PUR significantly enhanced ERK phosphorylation.

Accumulating evidence has demonstrated that oxidative stress plays an important role in the development of EBI (Ayer and Zhang, 2008; Duan et al., 2016). Following insults like hypoxia-ischemic or SAH, the production of reactive oxygen species (ROS) exceeds the ability of the endogenous anti-oxidant system, leading to oxidative stress and cell death (Wu et al., 2014; Zhang et al., 2016). Excessive production of ROS results in protein breakdown, lipid peroxidation, and DNA damage, leading to neuronal damage, BBB disruption, cellular apoptosis, and endothelial injury (Ayer and Zhang, 2008). Given the pathogenic impact of oxidative stress, therapeutic strategies aimed to blunt the processes are considered as an effective way to confer neuroprotection in SAH. A recent study demonstrated that PUR protected hippocampal neurons against injury through inhibition of oxidative stress (Peterson and Turnbull, 2012). In the present study, we found that the SAH insult increased MDA levels accompanied with decreased Shh, Gli-1 expression, and up-regulated Ptch expression, while PUR administration reversed MDA levels and Shh signaling molecules. Moreover, the neuroprotection of PUR on SAH was blocked by Smo antagonist Cyc. These results indicate that oxidative stress may be involved in an impaired Shh pathway following SAH, leading to neuronal damage and neurological deficits. Anti-oxidative strategies aimed at restoring the endogenous Shh pathway may offer an effective way to promote neurological function.

Nrf2 has been shown to be an important antioxidant protection in various CNS diseases, including SAH (Chen et al., 2011; Wu et al., 2014), traumatic brain injury (Jin et al., 2008), and neurodegenerative disorders (van Muiswinkel and Kuiperij, 2005). Under the pathological conditions, such as ROS, Nrf2 rapidly translocates from the cytoplasm to the nucleus. In the nucleus, Nrf2 binds to antioxidant-response element (ARE) and then activates ARE-dependent gene expression, such as heme oxygenase-1 (HO-1), NAD(P)H:quinone oxidoreductase 1, glutamate-cysteine ligase, glutathione peroxidase (Magesh et al., 2012). In the CNS, HO-1 was reported to be active in protecting cells exposed to oxidizing agents by catalyzing the oxidation of heme to biologically active products, including carbon monoxide, biliverdin, and ferrous iron (Syapin, 2008). Therefore, pharmacological activation of Nrf2/HO-1 system is critical for the protection of cells exposed to oxidative stress insults related to SAH (Chen et al., 2011). In the present study, we found that SAH induced a significant increase in Nrf2 protein levels in the PFC. The levels of Nrf2-regulated gene products, HO-1 was also up-regulated at 48 h after SAH, reinforcing previous report (Chen et al., 2011). Importantly, administration of PUR attenuated MDA levels and reduced the EBI in SAH rats, associated with an increase in the Nrf2 and HO-1 expression. However, the specific mechanism of activation of the Nrf2/HO-1 system by PUR has not been completely unknown, which needs to be investigated in further studies.

**Figure 9:**
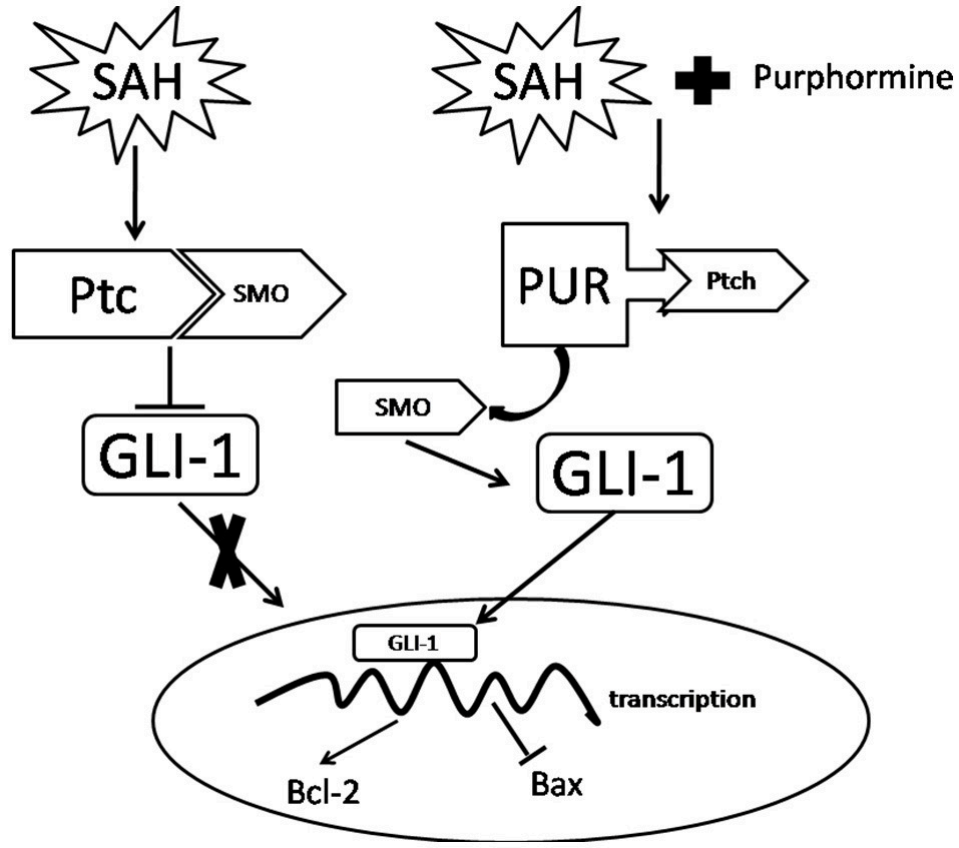
**A potential process illustrates the effects of PUR on early brain injury following SAH and the underlying mechanisms**. Briefly, treatment with PUR activated Ptch and caused Smo release. Then Gli-1 translocated to the nucleus, further up-regulated Bcl-2 expression and down-regulated Bax expression.

It should be noted that as recently researches indicated in the experimental SAH model, the Shh pathway (Shh, Gli-1, and Ptch) was up-regulated after 24 and 48 h injury, and Cyc administration at 10 mg/ml aggravated brain injury in SAH (Li et al., 2013). However, Zuo et al. showed that the protein of Shh was decreased at 24 h post-SAH (Zuo et al., 2015). In the study, we also found that at 48 h after SAH insult, Shh and Gli-1 expression were down-regulated. Notably, the expression of Ptch was up-regulated at 48 h post-SAH. Administration of Cyc alone at 1 mg/ml did not affect brain injury in SAH, while co-treatment with Cyc blocked the neuroprotective effects of PUR on SAH (Figure 9). There are a number of reasons for the inconsistencies in Shh pathway change after SAH insult. The different stimulating condition used in these studies maybe the main reason for these discrepancies, such as SAH model, the dose of Cyc administration.

However, our study has several limitations. Firstly, the cellular location of Shh, Gli-1, and Ptch, and the underlying mechanism of these proteins in SAH were not investigated. In addition, PUR may have other protective effects against EBI that were not evaluated in this study, such as anti-inflammatory and immunomodulatory properties. Lastly, only the tissue in PFC was analyzed, however, other brain regions, for example the hippocampus have increased neuronal apoptosis following SAH insult. Thus, the neuroprotective properties of PUR in SAH may also occur in other brain regions. Therefore, further studies are necessary.

In conclusion, we demonstrated that the Shh signaling agonist PUR plays a neuroprotective property during EBI after SAH insult and suggested PUR as a potential candidate in SAH therapy. The action of PUR was mediated in part by its anti-apoptotic and anti-oxidant mechanism, up-regulating phospho-ERK levels, mediating Shh signaling molecules in the PFC.

## Author contributions

GL and ZW were responsible for study design, data interpretation, and writing of the manuscript; QH and TL performed the experiments and analyzed data; LW, SL, XB, YX, and TZ were involved in setting up the animal model; HX was involved in manuscript editing. All authors read and approved the final manuscript.

## Funding

This work was supported by funding from National Natural Science Foundation of China (No. 81671213, 81571284); The Fundamental Research Funds of Shandong University (2015JC008).

### Conflict of interest statement

The authors declare that the research was conducted in the absence of any commercial or financial relationships that could be construed as a potential conflict of interest.

## Abbreviations

CNS: central nervous system
Cyc: cyclopamine
DAPI, 4′: 6-diamidino-2-phenylindole dihydrochloride
ERK: extracellular signal-regulated kinase
HO-1: heme oxygenase-1
MAPK: mitogen-activated protein kinase
MDA: malondialdehyde
Nrf2: NF-E2-related factor 2
PBS: phosphate buffered saline
PFC: prefrontal cortex
Ptch: Patched
PUR: purmorphamine
ROS: reactive oxygen species
RT-PCR: reverse transcription-polymerase chain reaction
Shh: sonic hedgehog
Smo: Smoothened.
